# Antiretroviral drug exposure in lymph nodes is heterogeneous and drug dependent

**DOI:** 10.1002/jia2.25895

**Published:** 2022-04-19

**Authors:** Elias P. Rosen, Claire Deleage, Nicole White, Craig Sykes, Catherine Brands, Lourdes Adamson, Paul Luciw, Jacob D. Estes, Angela D. M. Kashuba

**Affiliations:** ^1^ Division of Pharmacotherapy and Experimental Therapeutics University of North Carolina at Chapel Hill Chapel Hill North Carolina USA; ^2^ AIDS and Cancer Virus Program Frederick National Laboratory for Cancer Research Leidos Biomedical Research, Inc. Frederick Maryland USA; ^3^ Department of Pathology Microbiology & Immunology School of Veterinary Medicine University of California Davis Davis California USA; ^4^ Vaccine and Gene Therapy Institute Oregon Health & Science University Beaverton Oregon USA; ^5^ Oregon National Primate Research Center Oregon Health & Science University Beaverton Oregon USA

**Keywords:** antiretroviral, HIV, imaging, lymph node, mass spectrometry, tissue

## Abstract

**Introduction:**

HIV reservoirs and infected cells may persist in tissues with low concentrations of antiretrovirals (ARVs). Traditional pharmacology methods cannot assess variability in ARV concentrations within morphologically complex tissues, such as lymph nodes (LNs). We evaluated the distribution of six ARVs into LNs and the proximity of these ARVs to CD4^+^ T cells and cell‐associated RT‐SHIV viral RNA.

**Methods:**

Between December 2014 and April 2017, RT‐SHIV infected (SHIV+; *N* = 6) and healthy (SHIV–; *N* = 6) male rhesus macaques received two selected four‐drug combinations of six ARVs over 10 days to attain steady‐state conditions. Serial cryosections of axillary LN were analysed by a multimodal imaging approach that combined mass spectrometry imaging (MSI) for ARV disposition, RNAscope in situ hybridization for viral RNA (vRNA) and immunohistochemistry for CD4^+^ T cell and collagen expression. Spatial relationships across these four imaging domains were investigated by nearest neighbour search on co‐registered images using MATLAB.

**Results:**

Through MSI, ARV‐dependent, heterogeneous concentrations were observed in different morphological LN regions, such as the follicles and medullary sinuses. After 5–6 weeks of infection, more limited ARV penetration into LN tissue relative to the blood marker heme was found in SHIV+ animals (SHIV+: 0.7 [0.2–1.4] mm; SHIV–: 1.3 [0.5–1.7] mm), suggesting alterations in the microcirculation. However, we found no detectable increase in collagen deposition. Regimen‐wide maps of composite ARV distribution indicated that up to 27% of SHIV+ LN tissue area was not exposed to detectable ARVs. Regions associated with B cell follicles had median 1.15 [0.94–2.69] ‐fold reduction in areas with measurable drug, though differences were only statistically significant for tenofovir (*p* = 0.03). Median co‐localization of drug with CD4^+^ target cells and vRNA varied widely by ARV (5.1–100%), but nearest neighbour analysis indicated that up to 10% of target cells and cell‐associated vRNA were not directly contiguous to at least one drug at concentrations greater than the IC50 value.

**Conclusions:**

Our investigation of the spatial distributions of drug, virus and target cells underscores the influence of location and microenvironment within LN, where a small population of T cells may remain vulnerable to infection and low‐level viral replication during suppressive ART.

## 1 INTRODUCTION

Antiretroviral (ARV) therapy suppresses HIV replication below detection in plasma, yet cannot eradicate virus from circulating cells [1, 2] and tissues [3, 4, 5, 6] that form a reservoir to sustain the infection. Viral rebound can be seen in plasma within weeks of stopping ARV therapy [7, 8] even when treatment has been initiated rapidly following infection [9] or the size of the reservoir is small [10]. Rebound dynamics following analytical treatment interruption [11, 12] or voluntary treatment cessation near end‐of‐life [13] indicate that viral rebound can originate from varied anatomical compartments, including lymph nodes (LNs), which may serve as hubs for viral recrudescence [12, 13]. While there is growing evidence that clonal expansion represents a primary source for maintenance of virus harboured in tissue [14, 15, 16], there has been suggestion that active replication may occur in tissues with limited exposure to ARV drugs [17, 18].

The complex and highly compartmentalized architecture of LNs can be altered further by HIV pathogenesis [19, 20] and may result in altered penetration of drug [21] contributing to privileged anatomic regions like the B cell follicle [22] and its germinal centre where persistent viral transcription has been observed [23]. Significantly lower ARV concentrations have been measured in LNs relative to other lymphoid tissues [12, 18, 24], but cellular ARV concentrations remain uncertain since traditional methods for quantifying tissue ARV concentrations (tissue homogenates or isolated cells) [12, 18] either disrupt the native disposition of cells within a tissue or risk loss of intracellular drug during cell isolation [25].

Mass spectrometry imaging (MSI) represents an alternative method that can map drug distribution throughout an intact tissue sample [26, 27], resolving features at a scale that can be correlated to histochemical imaging to determine spatial relationships between the drug, target cells and virus [28]. These relationships have consequences not only for ascertaining whether ongoing replication is occurring, but also for design of curative strategies that rely on effective control of replicating virus [29].

Here, we utilize MSI to quantify the penetration of six ARVs relative to blood to evaluate diffusion of drug into LNs. We also combine infrared matrix‐assisted laser desorption electrospray ionization (IR‐MALDESI) MSI, in situ hybridization (ISH) and immunohistochemistry (IHC) to determine the proximity of ARVs relative to viral and target cell expression in axillary LNs of rhesus macaques (RM) infected with reverse transcriptase‐SIV expressing HIV‐1 envelope (RT‐SHIV).

## 2 METHODS

### 2.1 Animal study design

Detailed description of the animal study, conducted between December 2014 and April 2017, has been published previously [30]. Six male RM (*Macaca mulatta*) were inoculated intravenously with RT‐SHIVmac239 (SHIV+). At median 36 (34–42) days post inoculation, infected macaques were dosed daily for 10 days with emtricitabine and tenofovir (administered subcutaneously on the back to minimize scratching and irritation of injection sites) in combination with either oral maraviroc+atazanavir (four‐drug regimen denoted as FTMA; *N* = 3) or oral efavirenz+raltegravir (denoted as FTER; *N* = 3) to achieve pharmacokinetic steady‐state conditions for all agents. A matched cohort of healthy RM (SHIV–; *N* = 6) received the same ARV regimens. Dosing regimens, summarized in Table S1, and duration were chosen based on commonly used treatment doses for HIV infection in RMs to attain pharmacokinetic steady‐state conditions [31, 32]. Animals were euthanized and underwent necropsy approximately 24 hours after final ARV dose when LNs were collected and snap frozen in a methanol;dry ice bath. All animal experiments were performed in accordance with approved IACUC protocols from UNC‐Chapel Hill and the University of California at Davis.

### 2.2 Multimodal tissue imaging

Serial sections of 10 μm thickness were cut from snap frozen LN tissues by cryostat (Leica Biosystems, Buffalo Grove, IL) for parallel analysis by MSI (drug concentrations), IHC (CD4^+^ T cells and collagen) and ISH (viral RNA), respectively (see schema in Figure S1).

#### 2.2.1 MSI

ARV distribution was assessed by IR‐MALDESI MSI as previously described [26, 27, 33]. Briefly, tissue sections were ablated with an infrared laser such that each volumetric pixel, or voxel, represents 100×100×10 μm^3^. Desorbed material was ionized by an electrospray before chemical analysis by a Thermo Fisher Scientific Q Exactive mass spectrometer (Bremen, Germany). Absolute quantification of ARV concentrations was achieved by spotting calibration standards of known drug concentration onto blank RM LN tissue (Bioreclamation IVT, Baltimore, MD) [26, 34]. Per‐voxel limits of detection were assessed based on the standard deviation of each analyte from replicate measurements of drug concentrations spotted onto blank tissue samples, and the slope of the calibration curve, with a signal‐to‐noise ratio of 3. MSI ARV quantification was validated using LC‐MS/MS methods described previously [27, 35]. LC‐MS/MS precision and accuracy of calibration standards (0.1–50 ng/ml for all ARVs) and QC samples were within 15%. Tissue concentrations were reported as ng/slice and converted to μg/g using an assumed tissue density of 1.06 g/ml, reflecting the average density of secondary lymphoid tissue [36].

#### 2.2.2 IHC

Frozen LN sections were fixed in Prefer for 15 minutes after equilibrating to room temperature. After rinsing in ethanol, slides were placed in distilled water then in tris‐buffered saline (TBS)+Tween 20. Staining was performed for CD4^+^ T cells (Abcam clone BC/1F6; 1:100 dilution) and collagen type I (COL1; Sigma‐Aldrich C2456; 1:500 dilution) in TBS+Tween and incubated over night at room temperature. Slides were rinsed in TBS+Tween and golden bridge‐ AP‐ polymer P1 rabbit was incubated for 30 minutes at room temperature. Slides were rinsed with TBS+tween then revealed using Fast Red chromogen for 10 minutes.

#### 2.2.3 ISH

RT‐SHIV RNA expression in each sample was evaluated using RNAscope ISH [37, 38]. Slides were first fixed in 4% paraformaldehyde at 4˚C for 15 minutes followed by dehydration with graded ethanol washes (50%, 70% and 100% ethanol for 5 minutes each). Fixed slides were incubated for 10 minutes at 40˚C with protease digestion solution from ACD (P3) diluted in sterile cold PBS at 1:10 and rinsed with double distilled water and incubated with SIVmac239 ACD probes for 2 hours at 40˚C. Slides were then washed in 0.5X ACD wash buffer and incubated in amplification reagents according to RNAscope 2.5 HD detection protocol before counterstaining slides with Hematoxylin.

#### 2.2.4 Image analysis

MSI image data associated with all ARVs and endogenous ions of interest were identified [39] and extracted using MSiReader [34]. All imaging data were imported into Matlab for analysis using the Image Processing Toolbox. Colocalization of ARV distributions with endogenous ions associated with distinct morphological areas pertaining to blood vessels and B cell follicles was evaluated to isolate the ARV response measured that was present within these discrete areas. Multi‐modal image co‐registration combining MSI with ISH or IHC was performed at matched spatial resolution of MSI data (pixel size: 100×100 μm). The methods for colocalization and co‐registration have been described elsewhere [28, 40], and further details of image analysis to assess ARV localization, identify cell‐associated viral RNA and conduct nearest neighbour proximity search are found in the Supplementary Material.

#### 2.2.5 Statistical analysis

Descriptive statistics (median [range]) of tissue concentrations were generated for each drug and total regimen. Comparisons within groups of ARV concentration or fractional coverage based on disease state were performed using Wilcoxon rank‐sum test. Within‐sample comparisons of follicle fractional coverage and whole‐section fractional coverage were performed using Wilcoxon signed rank test. Comparisons between groups of ARVs were performed using the Kruskal–Wallis test, applying the Bonferroni correction to account for multiple comparisons. *p*<0.05 was considered statistically significant.

## 3 RESULTS

### 3.1 Heterogeneous distribution of ARV drugs in LN tissue

**Figure 1:**
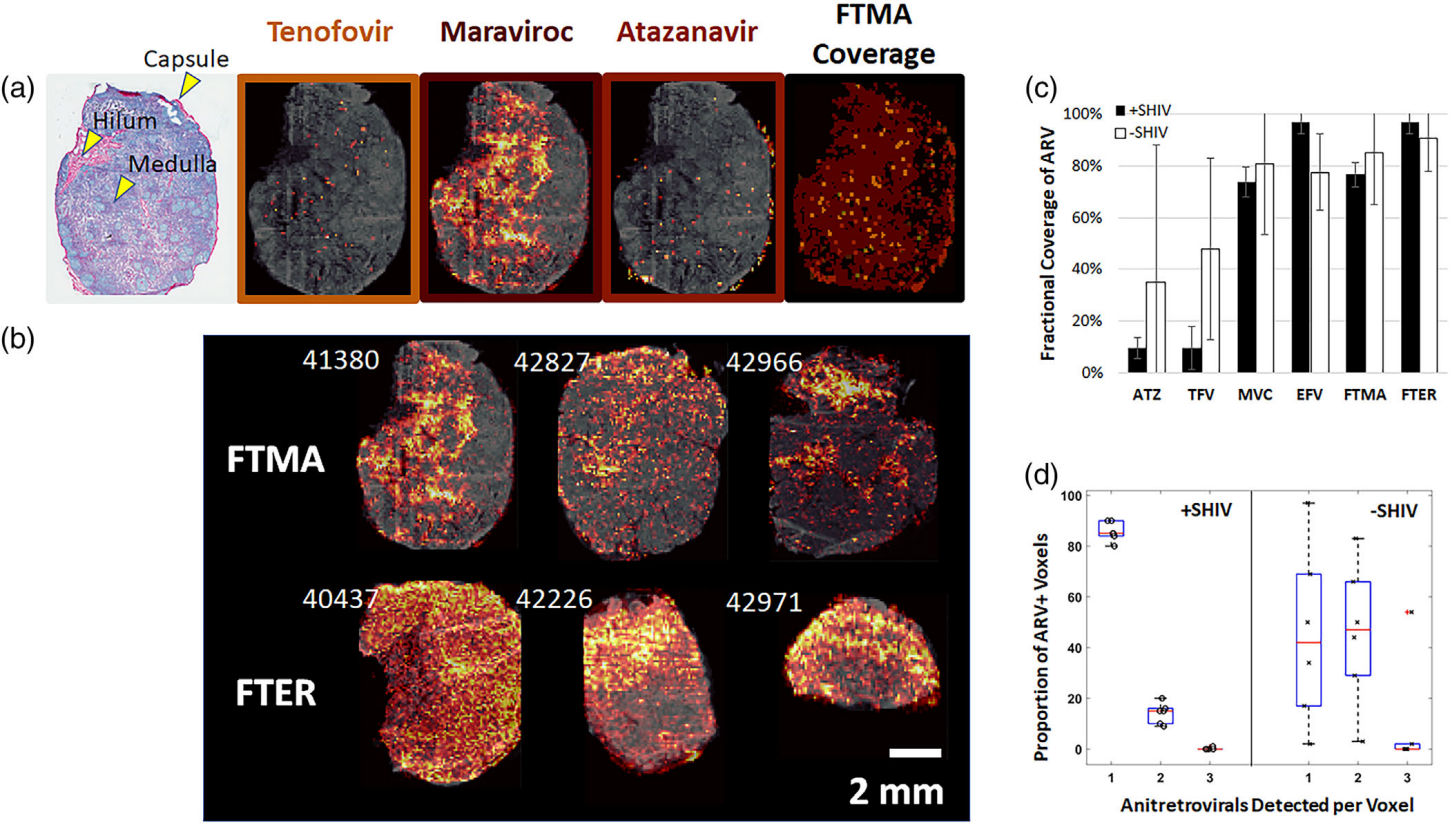
Heterogeneous ARV disposition in lymph nodes measured by IR‐MALDESI MSI. (a) Localization of individual ARVs within a representative single lymph node tissue section reflecting the FTMA regimen, with accumulation of drug represented by a colour scale ranging from lower drug abundance in black/dark red to higher drug abundance in yellow/white. Regions of accumulation for tenofovir, maraviroc and atazanavir measured within this representative sample can be observed relative to the tissue morphology shown in grey. Total drug exposure within the representative tissue section is demonstrated by overlaid binary maps for each individual drug. Collagen 1 stained brightfield image of an adjacent tissue section is provided for reference of section morphology. (b) Regimen‐wide drug exposure in each of the two dosing groups (emtricitabine + tenofovir + maraviroc + atazanavir, FTMA; emtricitabine + tenofovir + efavirenz + raltegravir, FTER) for all SHIV+ axillary lymph nodes, denoted by animal ID. (c) Proportional coverage of total lymph node tissue section area by ARVs alone and in combination for SHIV– (*n* = 6) and SHIV+ (*n* = 6) animals. (d) Extent of drug colocalization within lymph nodes of SHIV– and SHIV+ animals.

MSI revealed heterogeneous distribution of all detectable drugs throughout RM axillary LNs. Within‐tissue dynamic range (DR, log10 MSI signal abundance), reflecting the logarithmic ratio of highest to lowest detected MSI signal abundance, extended up to three orders of magnitude (1000‐fold range). Regions of accumulation, illustrated by representative FTMA and FTER sections in Figure 1a and Figure S2, respectively, varied by ARV: efavirenz distributed throughout tissue across all anatomic regions (DR_EFV_: 1.4 [1.2–2.1]); maraviroc preferentially accumulated in medullary sinuses and the paracortex (DR_MVC_: 2.2 [1.4–3.0]); atazanavir localized predominately in the capsule and subcapsular sinus (DR_ATZ_: 1.9 [1.6–2.9]); and tenofovir was distributed diffusely (and at very low concentrations) throughout tissue sections (DR_TFV_: 1.1 [0.4–2.8]). Raltegravir and emtricitabine concentrations in tissue were below IR‐MALDESI limits of detection (Table S2) and could not be quantified. Correlation and analysis of differences between LC‐MS/MS and IR‐MALDESI ARV concentrations, summarized in Figure S3, indicated that all measurements were linearly correlated (*R* ^2^>0.88) and within 95% confidence interval limits of agreement between the two methods. The pharmacologically active intracellular phosphorylated metabolites, tenofovir diphosphate and emtricitabine triphosphate, could not be quantified by IR‐MALDESI due to their degradation during sectioning [28]. LC‐MS/MS concentrations for all compounds, summarized in Figure S4, showed no significant differences between SHIV+ and SHIV– cohorts (*p* = 0.55 [0.10–1.00]). Relationships between IR‐MALDESI ARV concentration and proportional tissue coverage are shown in Figure S5, from which only tenofovir had greater proportional tissue coverage in SHIV– than SHIV+ cohorts (*p* = 0.04).

Regimen‐wide tissue coverage was evaluated through combination of individual ARV ion maps as illustrated in the color‐coded binary ion map in the right‐most cell of Figure 1a. Composite ion maps for each dosing group are shown for all SHIV+ (Figure 1b) and SHIV– (Figure S6) axillary LNs. Proportional coverage of LN tissue sections for ARVs, alone and in combination, in SHIV– and SHIV+ animals is illustrated in Figure 1c. ARV spatial distributions in SHIV+ tissues covered 87% [73–99%] of the tissue area, while ARVs in SHIV– tissues covered 96% [62–100%] (*p* = 0.55). In evaluating the amount of overlap between ARVs (Figure 1d), 15% [9–20%] of voxels sampled across SHIV+ tissue had more than one detectable ARV within the same voxel. In SHIV– tissue, overlap of drugs was more variable (58% [3–99%]), but differences with SHIV+ tissues were not statistically significant (*p* = 0.058).

**Figure 2:**
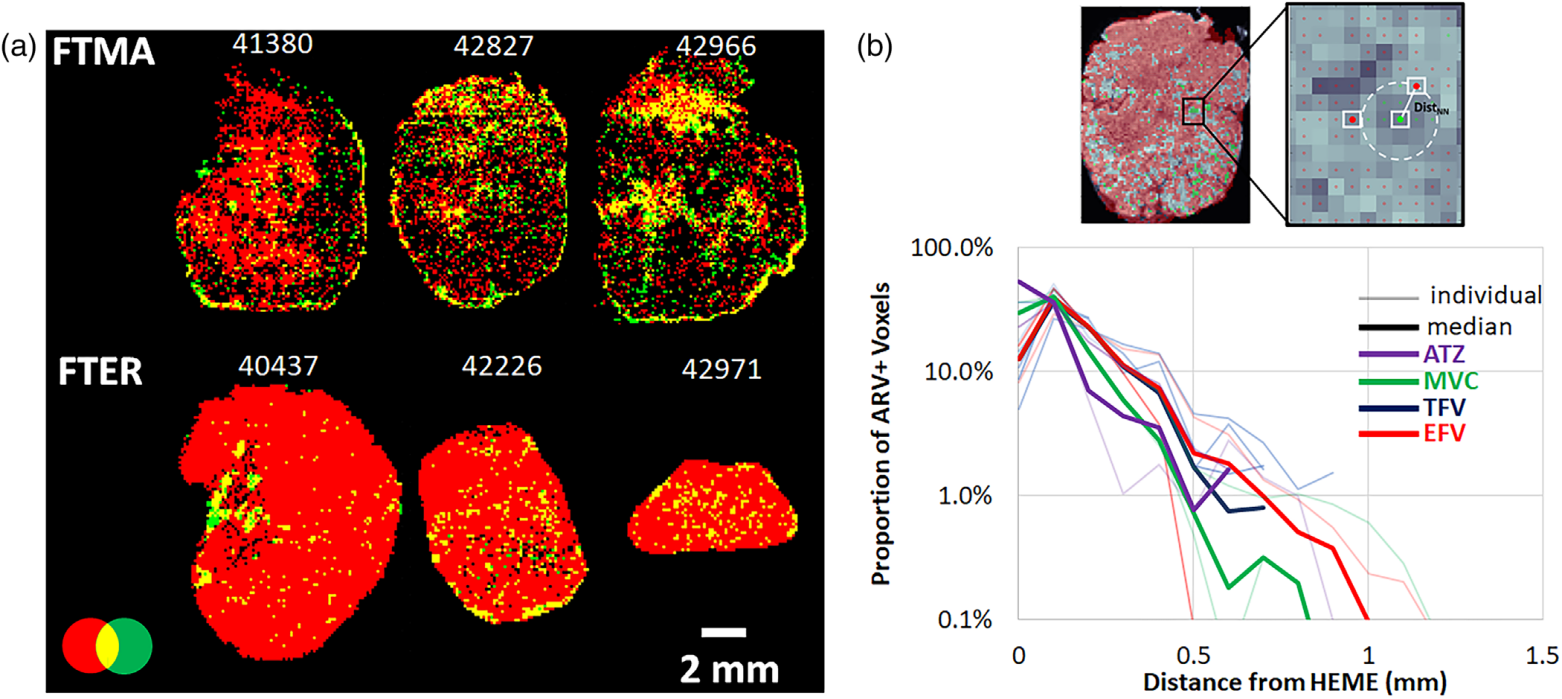
Penetration of antiretrovirals into lymph nodes relative to the blood marker heme in SHIV+ RM. (a) Binary overlay maps of regimen‐wide ARV exposure (red) and heme (green) with areas of overlap appearing yellow. (b) Distribution of observed shortest path distances from MSI sampling locations where heme was detected to each measured ARV as determined by nearest neighbour proximity search.

To calculate the penetration of drug into LN tissue relative to blood sources, we compared ARV distribution to the blood marker heme [41], which allowed the identification of blood vessels. Ion maps comparing the spatial distribution for ARVs and heme in SHIV+ animals are shown in Figure 2a. Colocalization between ARV response and heme, a median of 13–54% of the total response from each drug in all samples, was greatest for atazanavir and efavirenz in the SHIV+ and SHIV– cohorts, respectively. A nearest neighbour proximity search was used to compare shortest distance from heme to each ARV to quantify the diffusion range of ARVs from a blood vessel, summarized for the SHIV+ cohort in Figure 2b and all samples in Figure S7. The proportion of measured ARVs was greatest in voxels adjacent to heme and diminished with greater distance. All ARVs showed penetration greater than 0.5 mm into parenchymal tissue, with tenofovir and efavirenz disposition travelling to locations furthest from the blood vessel.

No indication of pathological changes to SHIV+ LN physical structure was found after ∼6 weeks of HIV infection (∼4 weeks of infection and ∼10 days of ARV treatment). The fractional coverage of heme (blood vessels) within LN tissue sections was similar in SHIV+ (12% [7–38%]) and SHIV– (12% [3–29%]) RM (*p* = 1.0). Additionally, no difference in COL1 deposition (Figure S8) was observed in SHIV+ (0.009 [0.006–0.02] COL1/nm^2^) and SHIV– (0.029 [0.015–0.041] COL1/nm^2^) RM (*p* = 0.11) that would signify progressive fibrosis at this stage of infection.

### 3.2 Colocalization of ARV drugs with CD4^+^ T cells and SHIV RNA expression

**Figure 3:**
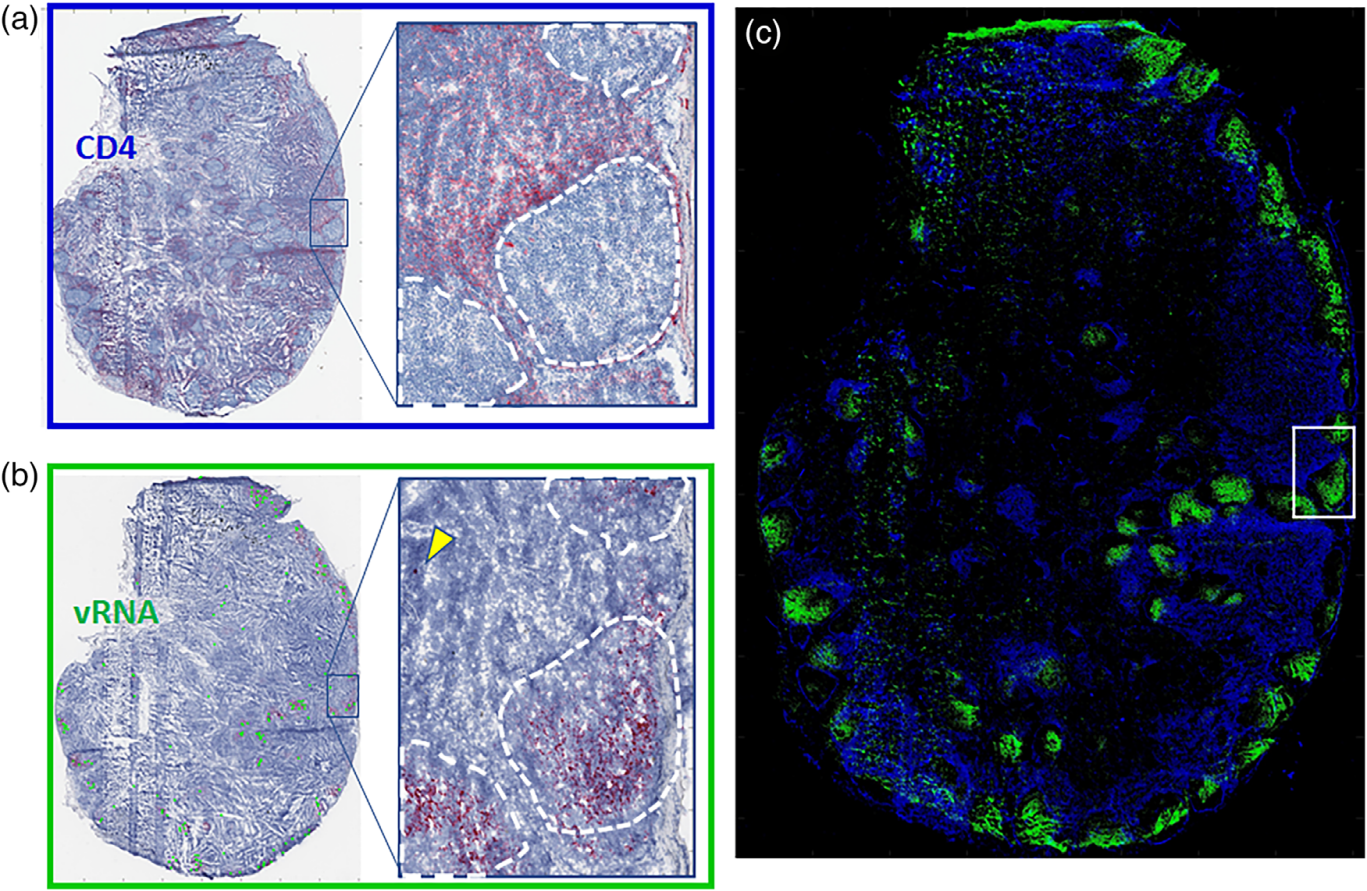
Immunohistochemistry and in situ hybridization analysis of adjacent tissue sections (representative sample matching Figure 1), with chromogenic Fast Red staining. (a) Disposition of CD4^+^ T cells was localized in the parenchymal T cell zone, with the inset showing the highest density surrounding B cell follicles. (b) Viral RNA expression was observed predominately within follicles, presumed bound to follicular dendritic cells, though productively infected vRNA^+^ cells remained identifiable (yellow arrow in inset). (c) Coregistered high‐resolution microscopy images of viral RNA (green) and CD4^+^ T cell (blue) distributions showing overlap between CD4 and cell‐associated viral RNA.

**Figure 4:**
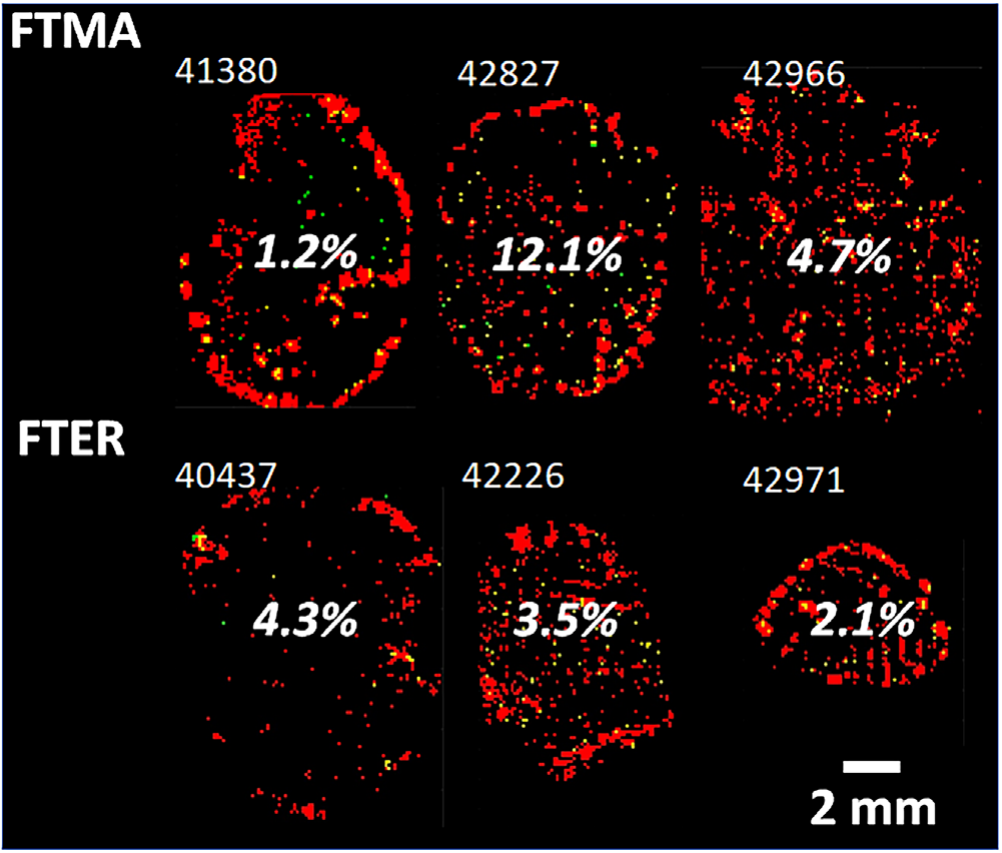
Proportion of viral RNA expression classified as being associated with productively infected cells. Total viral RNA response from in situ hybridization is shown in red and productively infected cells are shown in green.

CD4^+^ T cells were localized in the parenchymal T cell zone with highest density surrounding B cell follicles (Figure 3a). Representative SHIV RNA (vRNA) expression can be seen in Figure 3b. Most observable vRNA staining was found within follicles and bound to follicular dendritic cells (FDCs), based on the arrangement and density of virion within a germinal centre [42, 43, 44]. Productively infected vRNA^+^ cells remained identifiable in tissues following the 10 days of ARV treatment, which were differentiated from free virions algorithmically based on feature characteristics [37] (i.e. size, shape and intensity). Coregistered vRNA and CD4^+^ T cell distributions are shown in Figure 3c for comparison. Cell‐associated vRNA represented 3.9% [1.2–12.1%] of the total surface area of vRNA expression (Figure 4).

**Figure 5:**
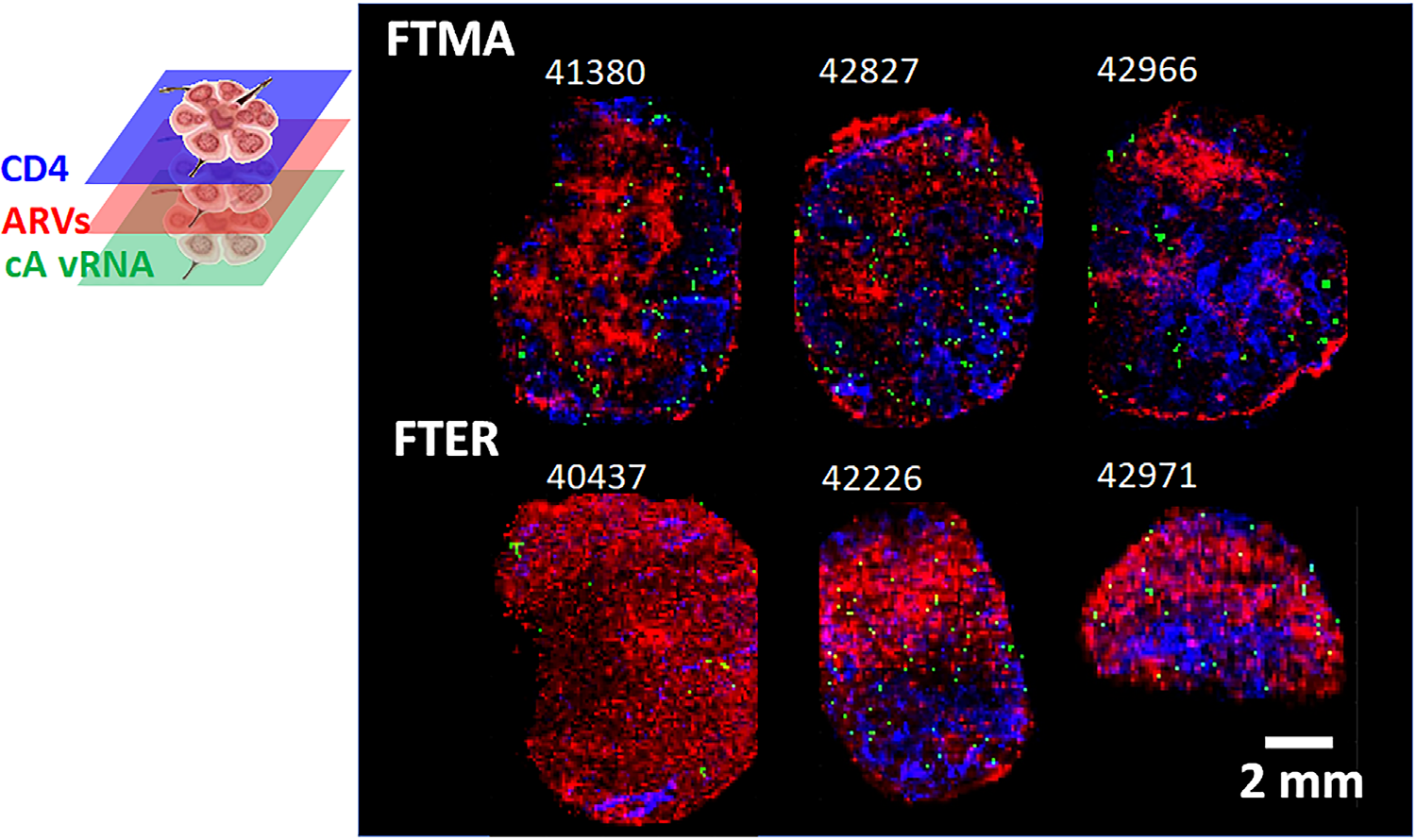
Colocalization of ARVs (red), CD4^+^ T cells (blue) and cell‐associated viral RNA expression (green) in SHIV+ RM lymph node tissue sections.

**Figure 6:**
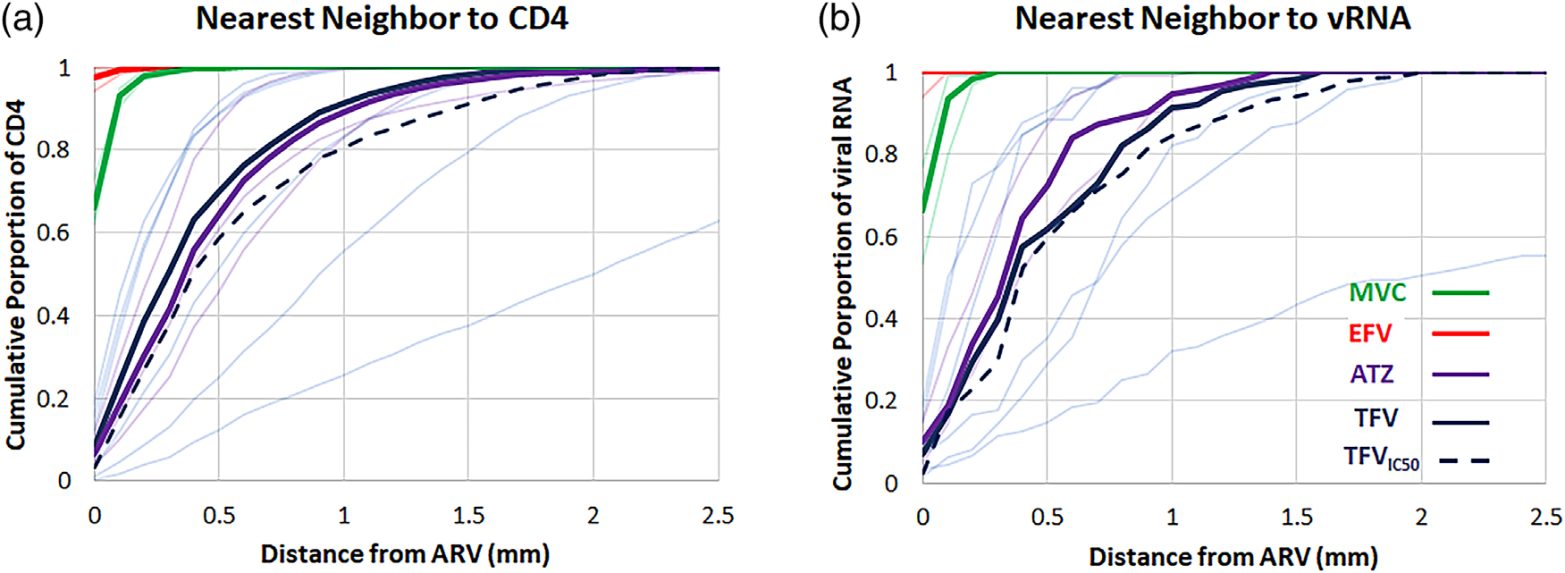
Nearest neighbouring detectable antiretroviral response to (a) CD4^+^ T cells and (b) productively infected cells containing viral RNA.

Colocalized maps of ARVs, corrected for potential blood contamination in locations where heme was measured, along with CD4^+^ T cell and cell‐associated vRNA expression at matched resolution are shown in Figure 5 for all SHIV+ animals. Drug exposure to regions of high CD4^+^ T cell and vRNA density varied within these samples. These results are summarized in Table 1 for individual drugs and multi‐drug regimens. Colocalization of target cells or viral RNA with detectable ARV concentrations was proportional to fractional coverage of ARVs across the tissue. Therefore, total regimen coverage was primarily driven by maraviroc and efavirenz distributions. Only efavirenz was observed to cover all cell‐associated vRNA expression and >98% of total CD4^+^ T cell distribution. However, when considering the proximity of target cells and virus to ARVs by nearest‐neighbour search (Figure 6, solid lines), >90% of all target cells and viral RNA expression associated with productive cells were adjacent (by one voxel) to a location containing measurable drug. Only one drug was measurable at locations adjacent to 58% [50–77%] of productively infected cells, which represents an upper‐bound on the proportion of cells potentially exposed to monotherapy based on MSI limits of detection. For drugs other than tenofovir, these locations reflect concentrations higher than in vitro 50% inhibitory concentrations (IC50) [45, 46, 47, 48]. The proportion of target cells or virus distribution colocalized with tenofovir concentrations exceeding the IC50 was more limited than exposure to any detectable tenofovir (Figure 6, dashed lines). In regions specifically associated with B cell follicles, a median 1.15 [0.94–2.69] ‐fold reduction in measurable drug relative to all anatomic regions across the tissue (Figure S9), though these differences were only statistically significant for tenofovir (*p* = 0.03).

**Table 1:** Colocalization of antiretrovirals with CD4 and cell‐associated vRNA

	Individual antiretroviral drugs				Multi‐drug regimen	
	TFV	ATZ	MVC	EFV	FTMA	FTER
CD4	8.0% (0.4–18.8%)	5.1% (3.6–11.2%)	63.4% (62.0–73.2%)	98.5% (94.1–99.9%)	66.5% (57.5–74.5%)	98.5% (94.2–100%)
cA vRNA	7.2% (1.6–19.2%)	10.0% (4.8–14.9%)	66.1% (53.3–77.0%)	100% (93.8–100.0%)	69.6% (56.7–82.8%)	100.0% (93.8–100.0%)

Note: Data are presented as median (range).

## 4 DISCUSSION

Our approach for MSI allowed both for the spatial mapping and quantification of ARV distribution within LNs. Regimen‐wide ARV exposure to axillary LNs was not uniform, as we have seen previously in mesenteric LNs [40] from the same RMs that had similar homogenate drug concentrations [35]. No two individual ARVs investigated were found to accumulate in the same patterns, with confinement in some cases to medullary sinus (maraviroc) or capsule (atazanavir), such that the proportion of LN cross‐section with at least one detectable ARV varied by four‐drug combination (FTER: >98%; FTMA: >82%). The extent to which ARV tissue coverage varied was drug specific. Emtricitabine and raltegravir were not detected by MSI and had the lowest LC‐MS/MS concentrations in LN [49], consistent with other studies [24, 50].

With ARV dynamic range up to three orders of magnitude within individual LNs, we characterized diffusion of drug from blood into tissue by comparing ARV disposition to the blood marker heme (a marker of blood vessels). Differences in nearest neighbour distance from measured ARVs to heme may reflect varied mechanisms of drug diffusion in LNs [21], since prior work did not find a relationship between drug transporter expression and ARV concentrations [35]. Median profiles of ARV concentration versus distance from heme differed based on infection status (Figure S6). Efavirenz and tenofovir had the greatest penetration distance in SHIV+ samples, while maraviroc and atazanavir had the greatest penetration distance in SHIV– samples. We considered the potential role of collagen as a means of modulating ARV tissue distribution. The RT‐SHIV backbone, SIVmac239, has been shown previously to induce progressive fibrosis in the early stages of infection, though development remained low and heterogeneous throughout the LN at 6 weeks post‐infection [38]. We found no difference in COL1 staining per unit area between SHIV– and SHIV+ tissues following 5–6 weeks of infection. Since viral load kinetics, and thus the magnitude of RT‐SHIV infection, is typically less than SIVmac239 [51], less pathology would be expected than typically seen in SIVmac239 and onset of fibrosis may be delayed. Even with no evidence of LN fibrosis at the time of our analysis, the differences in maximum penetration distance relative to heme based on infection status (SHIV+: 0.7 [0.2–1.4] mm; SHIV–: 1.3 [0.5–1.7] mm) suggest alterations to the microcirculation of drug based on infection status. At this early stage of infection, regimen‐wide coverage of LNs by ARVs or proportion of drug overlap was not significantly different by infection status. However, small‐scale changes in LN microcirculation may give rise to the lower penetration of ARVs in SHIV+ tissues relative to SHIV– tissues.

The observations of drug distribution in LN after 10 days of therapy reflect steady‐state pharmacokinetic conditions for the parent drugs. We designed this protocol to be at an early timepoint of infection and short duration of treatment, to evaluate drug distribution across productively infected RT‐SHIV cells that are still in the process of being controlled by treatment. Necropsy viral RNA load in plasma (1.5×10^3^ [1.5×10^1^–6.8×10^4^] SIV copies/ml) and LN (4.3×10^5^ [5.5×10^3^–4.1×10^6^] SIV copies/million cells) were consistent with an ARV effect before full‐suppression [52], with plasma viral load throughout infection and treatment shown in Figure S10.

Under these conditions, we observed active recruitment of CD4^+^ T cells within B cell follicles, where vRNA was predominately trapped within the FDC network. Previous studies estimated the average viral burst size to be 3–4 log10 virions/cell in a lifetime [53] and that virion production after T cell activation from individual proviruses varies by 10,000‐fold to 100,000‐fold [54], given a vast range of virion production per cell. We estimate that up to 99% of total vRNA was attributable to free virions. We confined our assessment of drug colocalization to CD4^+^ T cells and vRNA localized in productively infected cells, identified based on feature size and shape. Colocalization of ARVs with vRNA^+^ cells and CD4^+^ T cells indicated that not all target cells were directly exposed to measurable drug (FTMA: >56%; FTER: >93%). However, these images reflect a static picture of a dynamic system in which cells (uninfected and infected), virus and drug move throughout the LN. In consideration of this, we evaluated the proximity of target cells and virus to ARVs by nearest neighbour analysis (Figure 6). These results indicated that >90% of all target cells and vRNA expression associated with productive cells were adjacent to a location containing detectable drug. While multidrug colocalization was low in SHIV+ tissues, the extent to which continued dynamic movement may result in larger regions of effective synergistic activity [48, 55, 56] is not yet characterized. All of the ARVs considered in this study have demonstrated synergy with one of the accompanying administered drugs, suggesting efficacy at IC50 concentrations [48, 50, 57, 58] that have been adjusted where appropriate to account for protein binding in LN tissue [40].

Ongoing viral replication in the LN while on ART has been suggested to persist due to insufficient penetration of drug [17], though lack of evidence for viral divergence makes this hypothesis controversial [59, 60]. In considering both the direct and proximate colocalization of ARVs with target cells and vRNA^+^ cells, our results indicate that combination therapy provides a considerable protective bulwark to CD4^+^ T cells in LNs. While accumulation in discrete morphological regions of the LN was observed for individual drugs, the aggregate penetration of multiple drugs as part of a single regimen left only a small population of target cells in a localized unexposed pocket. Active replication occurring within these regions would likely be controlled rapidly by the buffer of ARVs in the immediate vicinity, or within CD4^+^ T cells moving out of these regions of higher ARVs. These results are consistent with ongoing viral replication in the LN being rare during treatment, such that the majority of the existing HIV burden observed within this tissue compartment [6] arises from other dominant sources, such as cellular proliferation [14] and latency [61]. However, transience and dynamics of such pockets remain to be evaluated longitudinally and these factors are potentially modulated by the combinations of ARVs that are selected for treatment as well as pathological alterations to lymphoid tissues that impact the microcirculation over the course of infection [19, 62], as our data suggest. Such pharmacological factors likely contribute to the interpatient variability in the degree of viral compartmentalization and location of rebound virus emergence in the limited number of individuals in whom viral dynamics have been investigated following treatment interruption [11, 13]. Further, our results have consequences for curative strategies like the shock‐and‐kill approach [29], which rely on ARV exposure to neutralize sustained viral replication following the activation of quiescent T cells to prevent repopulation of the viral reservoir, as well as underscoring the likely heterogeneous distribution of such cure therapies within tissues where reservoirs reside. While there is growing evidence that there are multiple anatomical sources of viral persistence that would need to be targeted for eradication, mapping relationships between LN antiviral drug penetration and target cells is critically important for the assessment of these curative strategies not only for individuals for whom the LN represents the source of HIV recrudescence following treatment interruption [11, 13], but more broadly because rapid trafficking of rebounding virus from disparate sources uses the LN as a hub for dissemination [13]. MSI offers a unique platform for evaluating distribution of ARV in tissues. These data, combined with biological information, such as cellular and viral distribution, can better inform the selection and dosing of tissue‐targeted therapeutics for maximal tissue exposure. While this approach entails an inherent tradeoff in sensitivity to preserve spatial information, and there is no currently known method of stabilizing intracellular phosphorylated metabolites of N(t)RTIs in tissue slices, MSI allows insight into the heterogeneous nature of drug distribution in tissues. Distributions in LNs of tenofovir and emtricitabine, which were administered to RMs subcutaneously, may differ from those in humans following oral dosing. This study did not allow us to evaluate the pharmacodynamic effect of localized ARV exposure on virus. However, we have created the multimodal imaging analysis framework to address this topic in future work that would also need to assess any changes to drug penetration with increased pathology. Inflammation accompanying greater pathology within the tissue may also increase the heterogeneity of tissue density, which may require revising an assumption of homogeneity of tissue density within a tissue section for quantitative analysis.

## 5 CONCLUSIONS

The spatial distributions of drug, immune cells and viral RNA expression (both vRNA^+^ cells and FDC‐bound virus) observed in this study of RM LNs provide contextual information underscoring the influence of location and microenvironment within a tissue compartment, otherwise lost by tissue homogenization or isolated cell analytical methods. The multimodal imaging approach developed for this work is capable of quantifying spatial relationships between drug and targets (blood, virus and T cells) and could be adapted to include host response as well. Its flexible framework allows ARVs to be evaluated individually or in aggregate and offers a tool to optimize pharmacokinetics and pharmacodynamics of HIV therapy.

## COMPETING INTERESTS

The authors have no competing interests.

## AUTHORS’ CONTRIBUTIONS

ER, PL and ADMK contributed to the design of the study. LA dosed the animals and collected tissues. NW and CS performed LC‐MS analyses. MM and GD performed the IHC staining and contributed to the analyses and interpretation of these data. CD, CB and JDE performed the IHC and ISH staining and contributed to the analyses and interpretation of these data. EPR and AK wrote the manuscript.

## Supporting information


**Supplementary Material**: Image analysis to assess ARV localization, identify cell‐associated viral RNA and conduct nearest neighbour proximity search.
