# Supplementary material for: Antiretroviral drug exposure in lymph nodes is heterogeneous and drug dependent
Source: J Int AIDS Soc. 2022 Apr 19;25(4):e25895. doi: 10.1002/jia2.25895 (PMC9018350; doi:10.1002/jia2.25895)
Supplement: Supplementary file 1 — Supplementary Material: Image analysis to assess ARV localization, identify cell‐associated viral RNA and conduct nearest neighbour proximity search. [file JIA2-25-e25895-s001.docx]

Supporting Information for

Antiretroviral drug exposure in lymph nodes is heterogeneous and drug-dependent

Elias P Rosen, Claire Deleage, Nicole White, Craig Sykes, Catherine Brands, Lourdes Adamson, Paul Luciw, Jacob D. Estes, Angela DM Kashuba

Correspondence to: [akashuba@unc.edu](mailto:akashuba@unc.edu)

**This PDF file includes:**

Fig. S1. Workflow for quantitative IR-MALDESI MSI analysis of RM axillary lymph node.

Fig. S2. Localization of individual ARVs within a representative FTER lymph node tissue sectionFig. S3. Comparison of antiretroviral drug quantitative analysis between IR-MALDESI MSI and LC-MS/MS.

Fig. S4. Cumulative antiretroviral drug concentrations in SHIV+ and SHIV- lymph nodes by LC-MS/MS.

Fig. S5. Antiretroviral drug coverage of lymph node tissues with respect to whole-section drug concentration.

Fig. S6. Antiretroviral drug distribution in SHIV+ and SHIV- lymph nodes.

Fig. S7. Heme nearest neighbor analysis for SHIV+ and SHIV- lymph node.

Fig. S8. Collagen 1 immunohistochemistry staining in SHIV+ and SHIV- lymph nodes.

Fig. S9. Follicle-specific antiretroviral signal abundance and fractional coverage.

Fig. S10. RM plasma viral load with respect to days of treatment.

Table S1. Antiretroviral Dosing of Rhesus Macaques.

Table S2. IR-MALDESI MSI Dynamic Range and Limits of Detection.

**Image Analysis**

Image co-registration and analysis was performed using the Matlab v.9.4 R2018a Image Processing Toolbox (Mathworks, Natick, MA). To isolate the proportion of ARV penetration in the lymph node that was associated with regions specific to an endogenous marker or a microscopy-derived distribution of target cells or viral expression, a binary mask of the spatial distribution was created. An example of the conversion process of a spatial distribution to a binary mask can be seen in Figure S9. Matrix multiplication of this binary mask and each ARV ion abundance matrix yielded the isolated ARV response within the region defined by the mask. Spatial relationships between ARVs (individually and in combination), heme, CD4+ T cells, and viral RNA were evaluated by nearest neighbor proximity search co-registered images using the Matlab **knnsearch** function.

Applications of this approach within the current work are described below.

*ARV colocalization with heme*

The distribution of heme (*m/z* 616.1780) measured by MSI was chosen to delineate the presence of blood in lymph node tissue sections within vasculature or high endothelial venules. The binary mask created from the heme distribution in each tissue section was used to identify the sampling locations where ARVs were measured that represented the microvasculature network. The isolated ARV response at each of these sampling locations could then be subtracted from the original ARV distributions to create a revised map of drug penetration exclusively within tissue. The heme-corrected map associated with each ARV in a sample was used to assess spatial relationships between drug and target cells or viral expression.

*ARV colocalization with CD4 and viral RNA*

ISH and IHC microscopy images of viral RNA and CD4+ T cells, respectively, were color thresholded to isolate staining from counterstain. Cell-associated viral RNA expression was isolated from free virion response based on feature intensity, area, and shape eccentricity using the Matlab **regionprops** function and compared to annotations by expert hand of productively infected cells. Co-registered microscopy data was then downsampled by a factor of 206 [190-231] to match the spatial resolution of MSI data (pixel size: 100x100 μm). All image alignment was performed by manual selection of control points followed by rigid registration.

After image co-registration was performed, ARV was masked based on the distribution of CD4 or vRNA and was used to determine the proportion of total distribution of the cells or virus that co-localized with individual ARVs. Quantitative MSI data were used to determine median concentration of each ARV co-localized with a given variable based on signal abundance and the total ARV concentration per slice. To assess whether ARV penetration was theoretically sufficient to suppress viral replication in the long term, observed ARV concentrations were compared to the *in vitro* IC_50_ values for each.

*ARV colocalization with B cell follicles*

B cell follicles were identified initially from the combined isolated staining of viral RNA and CD4+ T cells. As described in the Results section, viral RNA was predominately present within the follicles trapped to the follicular dendritic cells IHC CD4 response and CD4+ T cells were present with highest density surrounding B cell follicles. Endogenous ions with preferential expression within the follicles were determined using the structural similarity (SSIM) algorithm within MSiReader using each of the downsampled viral RNA expression and inverted CD4+ expression (making the highest response within the follicle) as reference images. SSIM analysis identified 5 ions (m/z 368.3367, 407.3295, 673.5939, 707.5388, and748.6282) with highest measured signal abundance in patterns closely matching those of the viral RNA expression and inverted CD4+ expression. A composite ion map representing the average of these 5 ions was used to create a binary mask delineating the bounds of the follicles, as shown in Figure S8. Follicle locations defined by this process agreed with hand annotations of follicles from microscopy images. The composite binary mask associated with each sample section was used to isolate ARV response within sampling locations that correspond to B cell follicles.

**
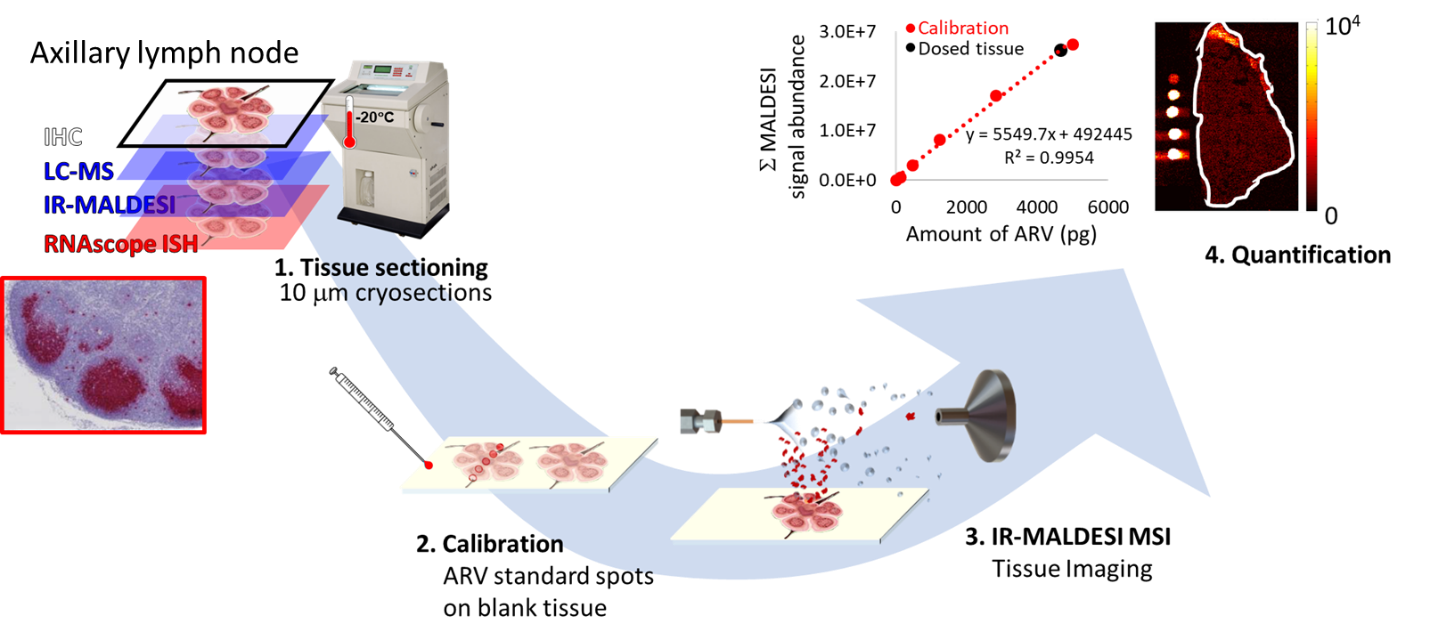
 Figure S1. Workflow for quantitative IR-MALDESI MSI analysis of RM axillary lymph node.**


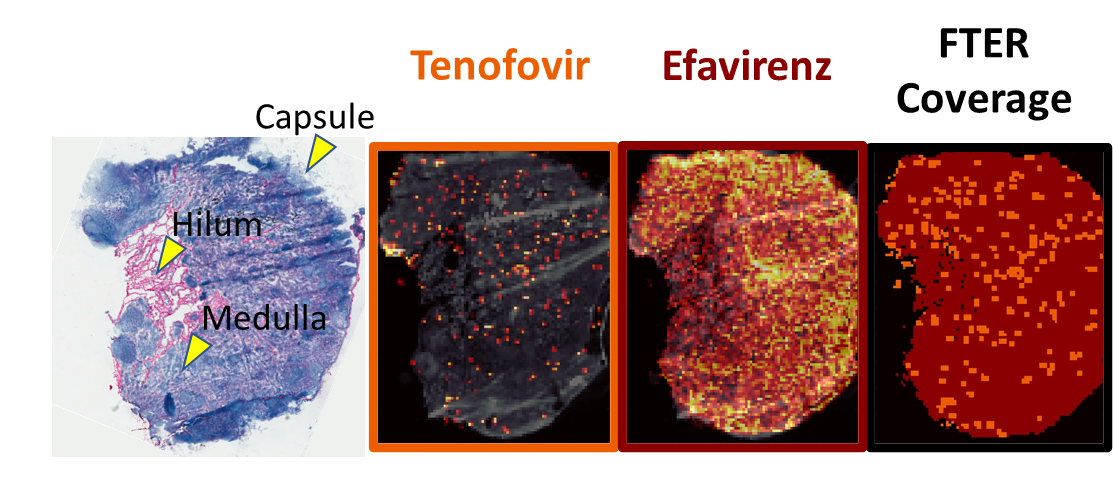


**Fig. S2. Localization of individual ARVs within a representative FTER lymph node tissue section.** Accumulation of drug is represented by a color scale ranging from lower drug abundance in black/dark red to higher drug abundance in yellow/white. Regions of accumulation for tenofovir and efavirenz measured within this representative sample can be observed relative to the tissue morphology shown in gray. Total drug exposure within the representative FTER tissue section is demonstrated by overlaid binary maps for each individual drug. Collagen 1 stained brightfield image of an adjacent tissue section is provided for reference of section morphology


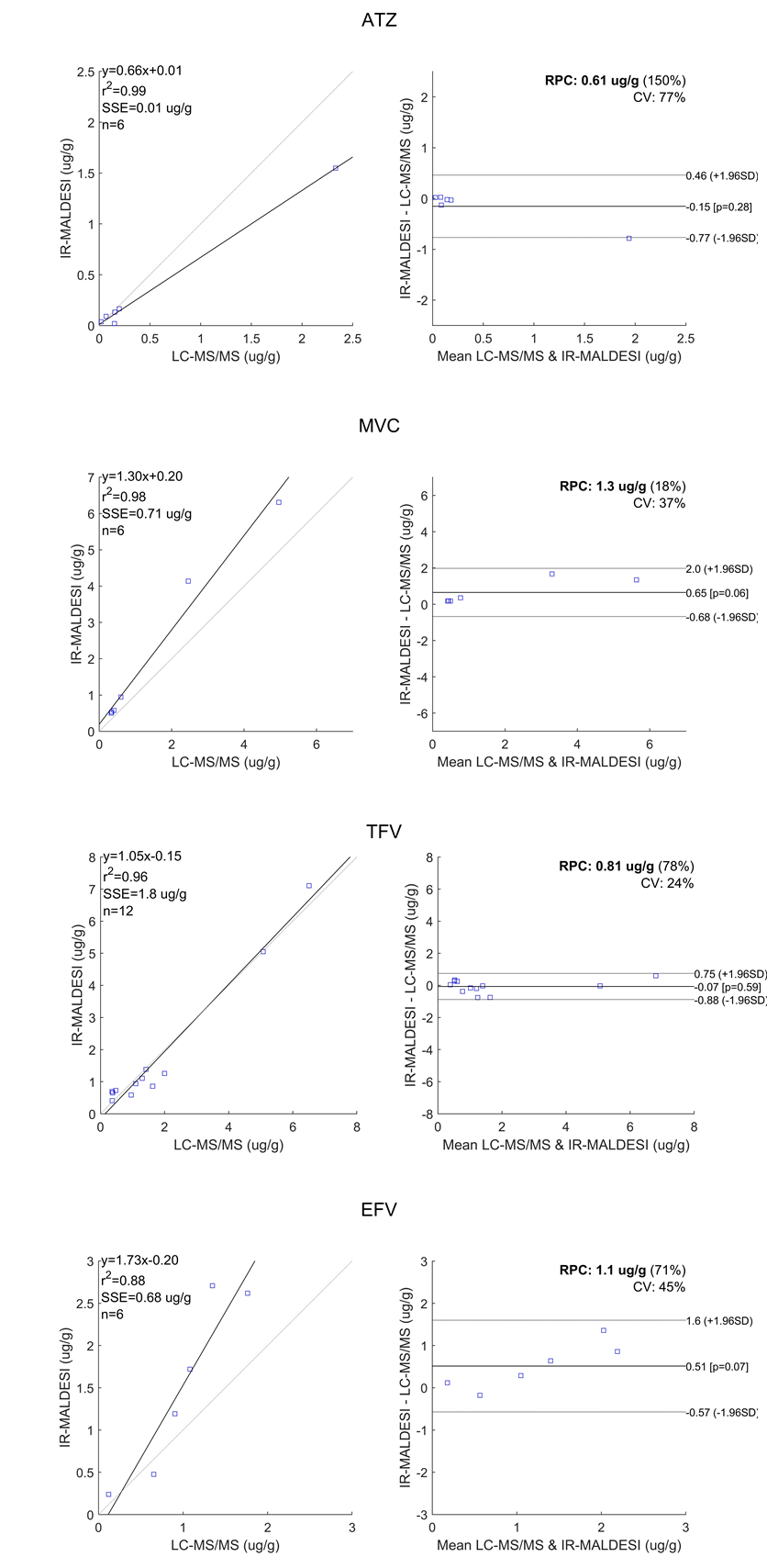


SSE - sum of squared error

RPC - reproducibility coefficient (1.96*SD)

Fig. S3. Comparison of antiretroviral drug quantitative analysis between IR-MALDESI MSI and LC-MS/MS.

**
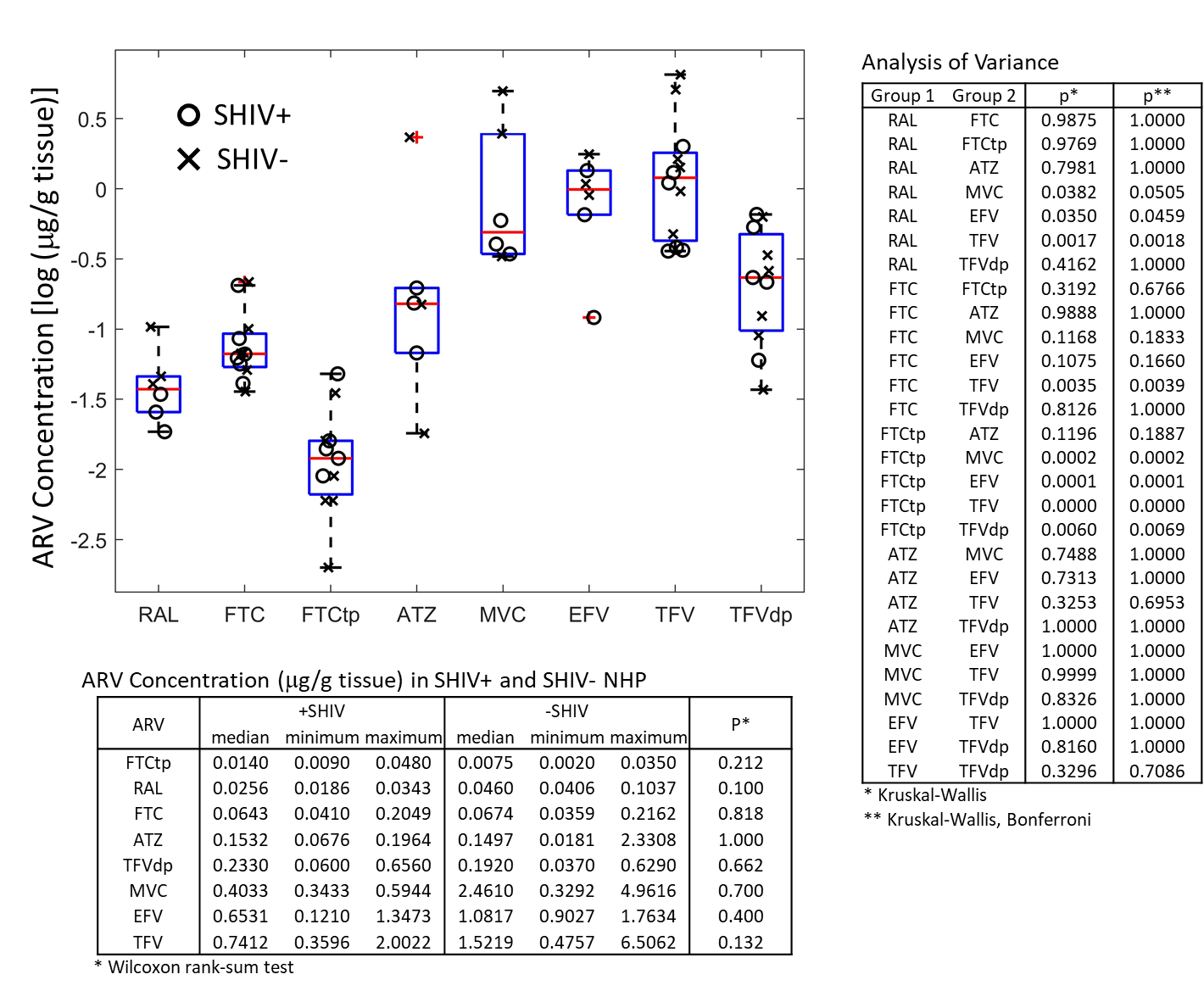
**

Fig. S4. Cumulative antiretroviral drug concentrations in SHIV+ and SHIV- lymph nodes by LC-MS/MS.

**
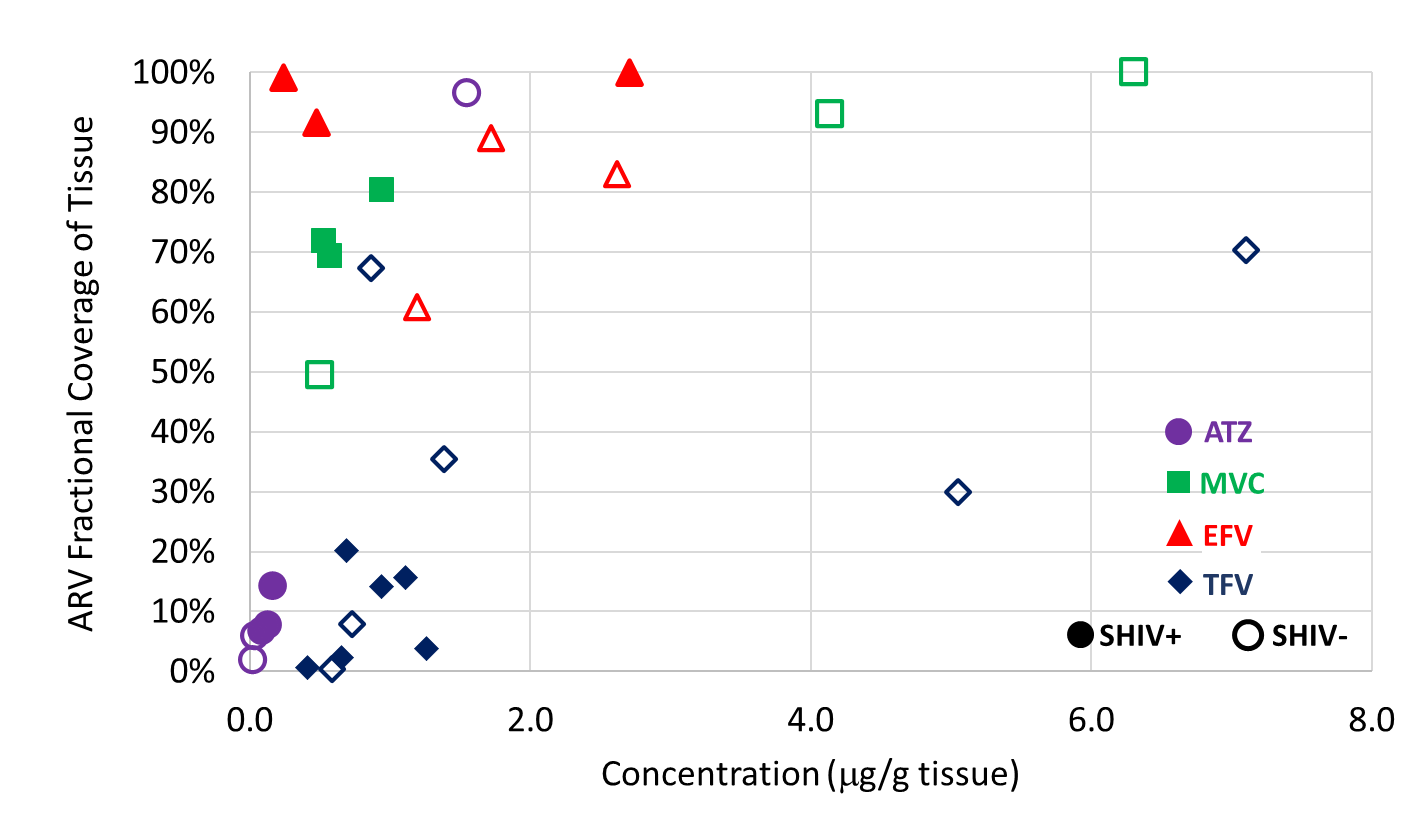
**

**Fig. S5. Antiretroviral drug coverage of lymph node tissues with respect to whole-section drug concentration.**

This figure compares the total concentration of antiretroviral drugs measured in all individual lymph node tissue sections (SHIV+: closed symbols; SHIV-: open symbols) by IR-MALDESI with the proportional coverage of the tissue section by each measured drug. The relationship between fraction of tissue area with detectable drug and total drug concentration was drug-specific.

**
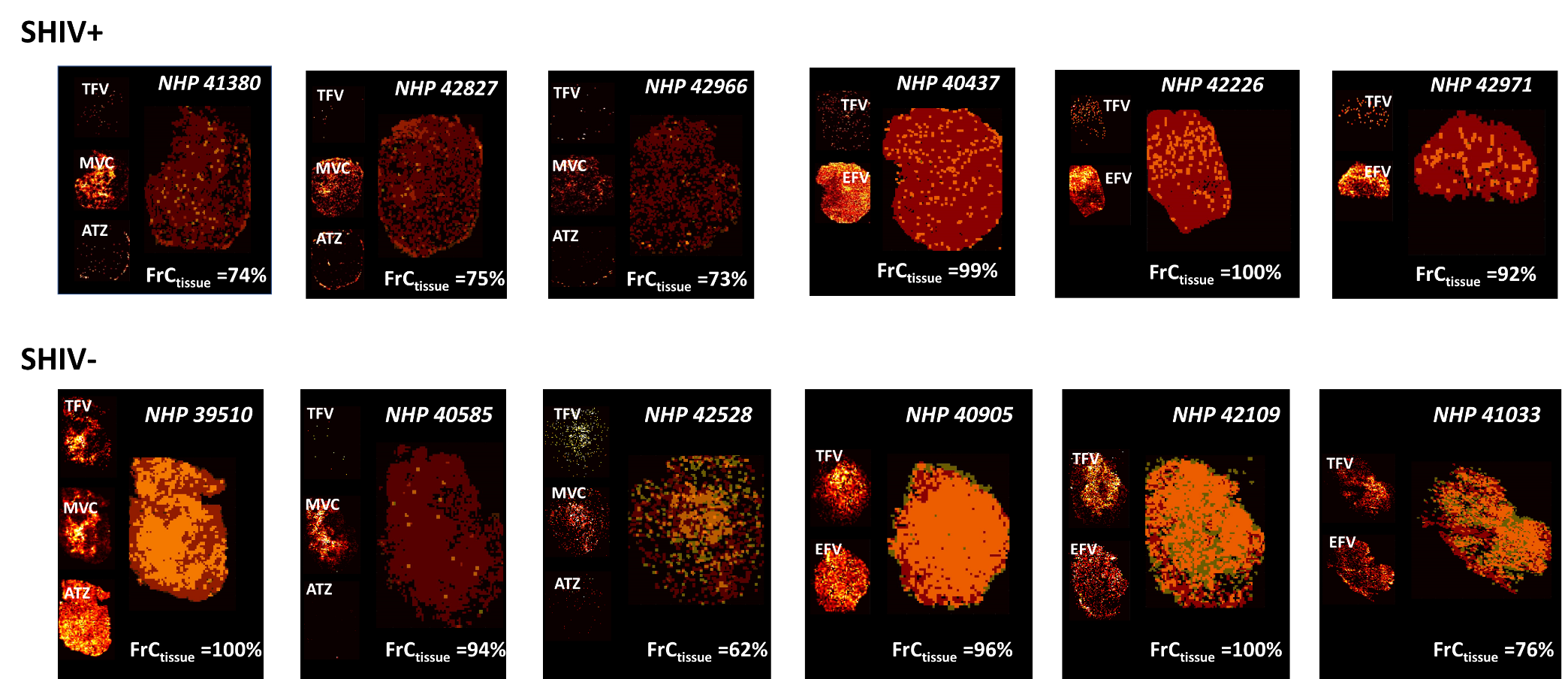
**

**Fig. S6. Antiretroviral drug distribution in SHIV+ and SHIV- lymph nodes.**

For each lymph node, total drug exposure is shown by the large overlaid binary maps of each detected ARV with the disposition of individual ARVs provided separately to the left. Fractional coverage (FrC) of the tissue by detected ARVs is indicated at the bottom of each group of images.


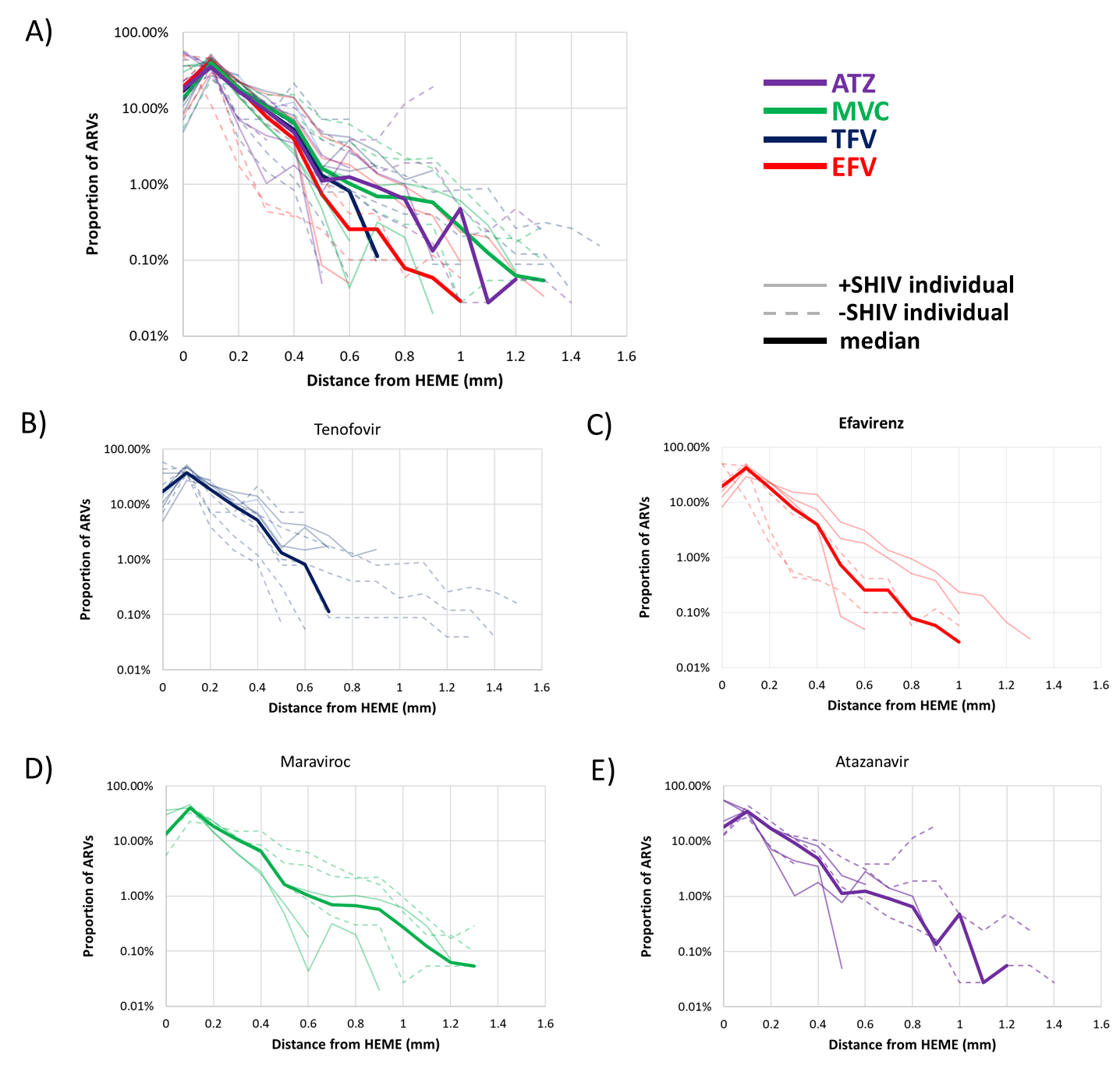


**Fig. S7. Heme nearest neighbor analysis for SHIV+ and SHIV- lymph node.**

Nearest-neighbor frequency distribution profiles are provided for individual animals (SHIV+: thin solid line; SHIV-: thin dashed line) and for the median response (thick solid line). Profiles for all detected ARVs are shown in (A). Profiles for each ARV are shown for: (B) tenofovir; (C) efavirenz; (D) maraviroc; and, (E) atazanavir.


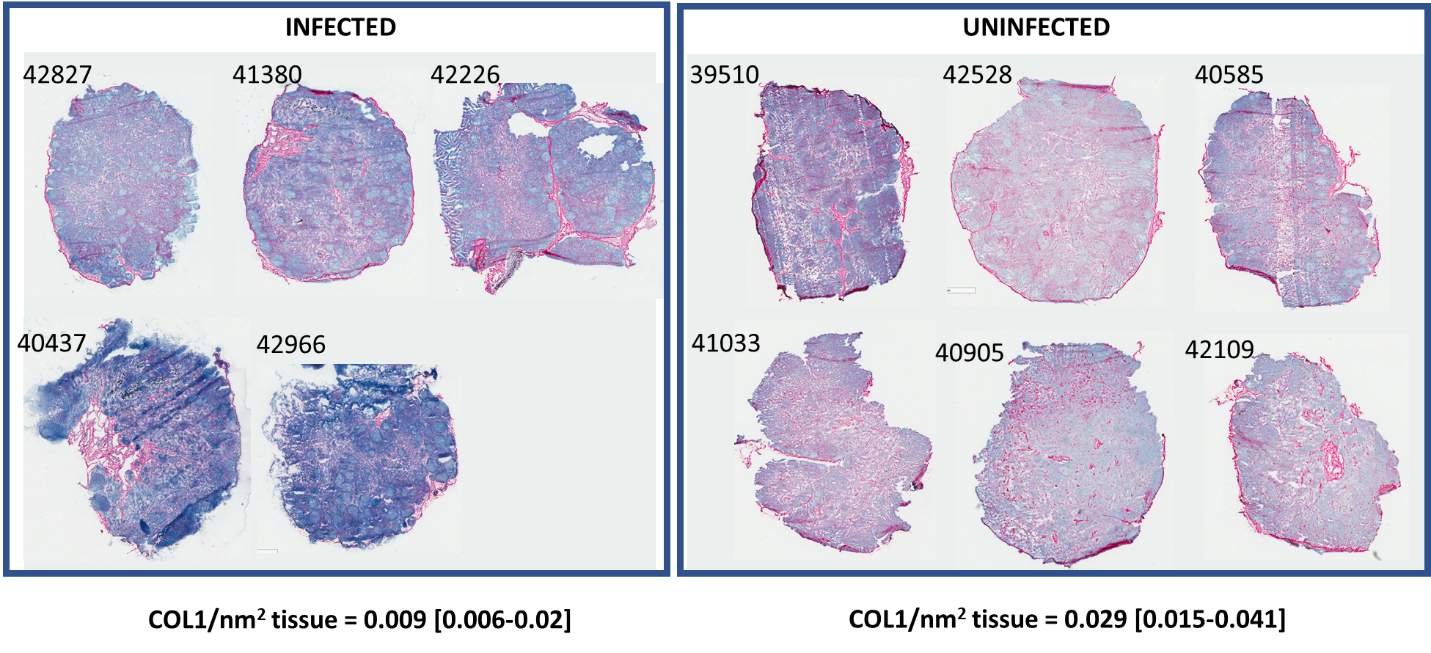


**Fig. S8. Collagen 1 immunohistochemistry staining in SHIV+ and SHIV- lymph nodes.**

**
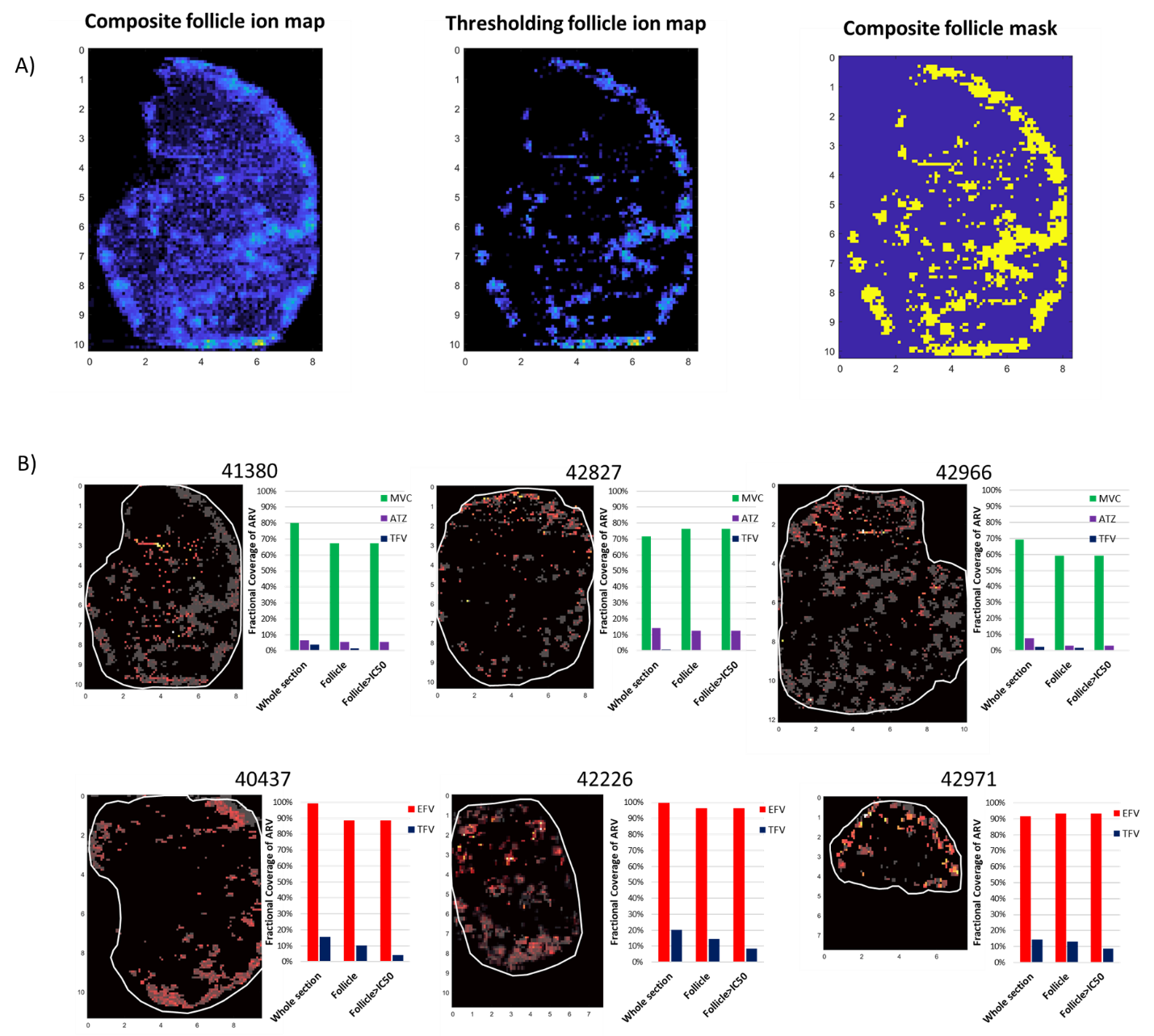
**

**Fig. S9. A) B cell follicle morphological masking based on composite response to upregulated endogenous lipids within the follicle of SHIV+ samples. B) Follicle-specific antiretroviral signal abundance and fractional coverage.**


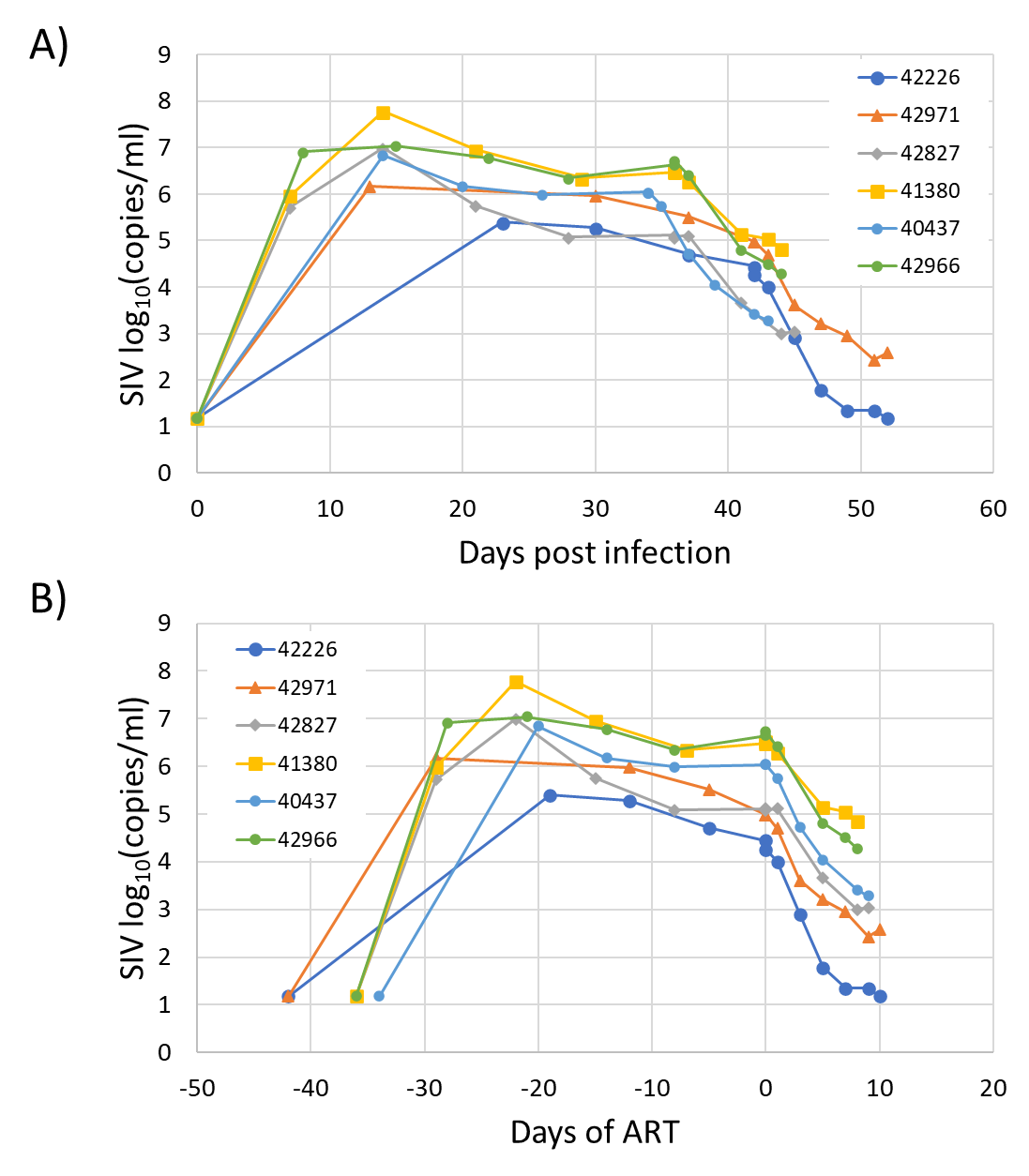


Fig. S10. RM plasma viral load with respect to A) days post infection and B) days of treatment.

Table S1. Antiretroviral Dosing of Rhesus Macaques.

| ^a^ log_10_(Signal Abundance) |
| --- |
| ^b^ Per-voxel limits of detection were assessed based on the standard deviation of each analyte from replicate measurements of blank samples and the slope of the calibration curve with signal-to-noise ratio of 3.  ^c^ protein-binding corrected |

Table S2. IR-MALDESI MSI Dynamic Range and Limits of Detection.
